# FGD-Gypsum Waste to Capture CO_2_ and to Recycle in Building Materials: Optimal Reaction Yield and Preliminary Mechanical Properties

**DOI:** 10.3390/ma17153774

**Published:** 2024-08-01

**Authors:** Virginia Moreno, Judith González-Arias, Jaime D. Ruiz-Martinez, Rafael Balart-Gimeno, Francisco Manuel Baena-Moreno, Carlos Leiva

**Affiliations:** 1 Institute of Materials Technology (ITM), Universitat Politècnica de València (UPV), Plaza Ferrándiz y Carbonell 1, 03801 Alcoy, Spain; rbalart@mcm.upv.es; 2 Inorganic Chemistry Department, Materials Sciences Institute, University of Seville-CSIC, 41004 Seville, Spain; jgonzalez15@us.es; 3 Department of Chemical and Environmental Engineering, School of Engineering, University of Seville, Camino de los Descubrimientos s/n, 41092 Seville, Spain; jruiz3@us.es (J.D.R.-M.); fbaena2@us.es (F.M.B.-M.)

**Keywords:** flue gas desulfurization, CO_2_ capture, calcium carbonate, waste valorization, building material

## Abstract

The use of waste to capture CO_2_ has been on the rise, to reduce costs and to improve the environmental footprint. Here, a flue gas desulfurization (FGD) gypsum waste is proposed, which allows us to obtain a CaCO_3_-based solid, which should be recycled. The CO_2_ capture stage has primarily been carried out via the direct carbonation method or at high temperature. However, a high energy penalty and/or long reaction times make it unattractive from an industrial perspective. To avoid this, herein an indirect method is proposed, based on first capturing the CO_2_ with NaOH and later using an aqueous carbonation stage. This allows us to capture CO_2_ at a near-ambient temperature, improving reaction times and avoiding the energy penalty. The parameters studied were Ca^2+^/CO_3_^2−^ ratio, L/S ratio and temperature. Each of them has been optimized, with 1.25, 100 mL/g and 25 °C being the optimal values, respectively, reaching an efficiency of 72.52%. Furthermore, the utilization of the produced CaCO_3_ as a building material has been analyzed. The density, superficial hardness and the compressive strength of a material composed of 10 wt% of CaCO_3_ and 90 wt% of commercial gypsum, with a water/solid ratio of 0.5, is measured. When the waste is added, the density and the mechanical properties decreased, although the compressive strength and superficial hardness are higher than the requirements for gypsum panels. Thus, this work is promising for the carbonation of FGD-gypsum, which involves its chemical transformation into calcium carbonate through reacting it with the CO_2_ of flue gasses and recycling the generated wastes in construction materials.

## 1. Introduction

The increasing accumulation of industrial waste and the rising levels of atmospheric CO_2_ are two of the pressing environmental challenges of our time [1,2]. As industrial activities expand, the production of side streams and emissions intensifies, exacerbating environmental degradation and contributing to climate change. Among these side streams, flue gas desulfurization gypsum (FGD-gypsum) is highlighted. FGD-gypsum is a result of flue gas desulfurization processes used in power plants and industrial facilities, and it currently represents a significant waste management issue. In 2020, global FGD-gypsum production is expected to reach 255 million tons, with Asia accounting for 55%, followed by Europe (22%), North America (18%), and the rest of the world (5%). Approximately 75% of this production is used in cement and concrete production and agriculture, mostly for the manufacture of gypsum boards [3]. Traditionally, FGD-gypsum is disposed of in landfills, which not only occupies valuable land space but also poses the potential risks of leaching and groundwater contamination [3,4,5,6].

On the other hand, the rising concentration of CO_2_ in the atmosphere, primarily due to fossil fuel combustion and deforestation, is driving global climate change [7,8]. Mitigating CO_2_ emissions is a major priority in the attempt to minimize global warming and its related repercussions, such as extreme weather events, sea level rise, and disruptions of ecosystems. In this context, innovative strategies to jointly address both waste management and CO_2_ capture are urgently needed [2,9,10].

One promising solution to “kill two birds with one stone” lies in the carbonation of FGD-gypsum, which involves its chemical transformation into calcium carbonate (CaCO_3_) through reacting it with the CO_2_ of flue gasses. This process not only converts a problematic waste material into a valuable product but also captures CO_2_ in a stable and solid form. This dual benefit positions FGD-gypsum carbonation as a potentially impactful approach to both waste reduction and CO_2_ mitigation.

In fact, previous works have investigated the carbonation of FGD-gypsum to produce CaCO_3_, most of them using the so-called direct carbonation [11,12,13]. However, this route is kinetically slow, which makes it unlikely for industrial applications [14]. Other previous works have proposed an indirect carbonation method, which consists of a first stage in which CO_2_ is absorbed by NaOH, and the formed Na_2_CO_3_ reacts later with an alkaline waste to precipitate CaCO_3_ [15,16,17]. Nonetheless, for the indirect carbonation of FGD-gypsum, there are many research questions that remain unanswered. Examples of these are: What is the optimal Ca^2+^/CO_3_^2−^ ratio for the reaction? How does the liquid to solid ratio influence the carbonation? What role does the temperature play here? Some of these parameters are key for the industrial development of this process. This work aims not only to answer these questions but also to go one step further. Our one step ahead consists of utilizing the produced CaCO_3_ as a building material, closing the circle from a circular economy perspective. 

**Figure 1:**
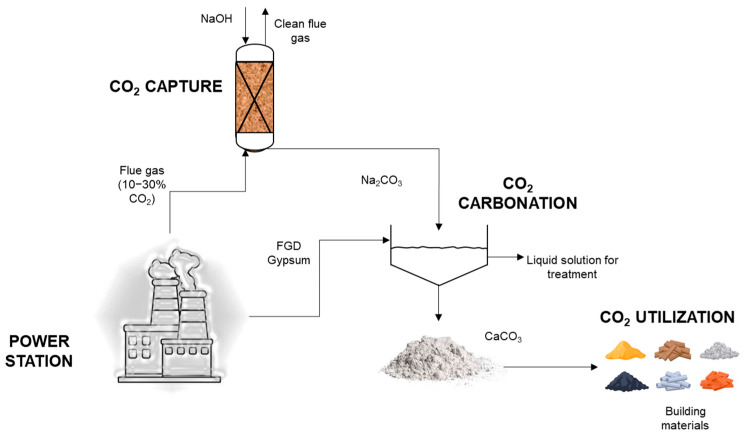
Schematic of the process from waste to building materials.

Calcium carbonate has been used as a mineral filler in gypsum panels [18], so that even industrial side streams containing these components (eggs [19], ladle furnace slag [20] and carbonated fly ash [21]) have been used successfully as an addition to gypsum products. The final objective of this work is to reuse the material after the capture of CO_2_, hence entailing a minimum solid waste generation. Figure 1 schematizes our whole idea.

To meet the pursued goal, our work is organized as follows. First, the materials used and the methodology employed are described. Secondly, the results regarding the reaction yield are explained in-depth for the three parameters mentioned above. A complete physicochemical characterization follows this section in which the formation of CaCO_3_ and the principal characteristics of the product are corroborated. The Results section ends by studying the compression strength and the bending strength of this material produced.

## 2. Materials and Methods

### 2.1. Materials

The chemical compounds employed in the experiments, such as Na_2_CO_3_, CaCO_3_ and NaOH, were supplied by PanReac-AppliChem (pure-grade, 99% purity). The desulfurized gypsum (FGD) used in this research was provided by DGC units in Compostilla (G-C) (Ponferrada; Spain).

A commercial gypsum (COMg) from the company AFIMOSA S.L. was also employed, in accordance with EN 13279-1 (CEN, 2009) standard [22].

Table 1 shows the chemical composition of FGD-gypsum and COMg and, as can be seen, the FGD-gypsum presents a high humidity. The reason for this is that the FGD-gypsum provided comes from a wet desulfurization unit. Moreover, both are composed mainly of calcium sulfate, although the SO_3_ content of the COMg is lower than FGD-gypsum, because the commercial gypsum presents some impurities during its extraction process.

### 2.2. Methodology

#### 2.2.1. Experimental Procedure

To perform the experimental procedure, laboratory-scale batches were carried out in a 250 mL beaker and placed on a stirring plate at room temperature. The procedure begins by diluting 5 g of Na_2_CO_3_ in 100 mL of distilled water and maintaining constant stirring (500 rpm). Once the dissolution was completed, the desulphurized gypsum residue was added slowly for approximately 5 min. The pH of the dissolution is important, since it provides us with information about the compounds that may be present and the timeframe of the reaction [15]. Therefore, to corroborate the end of the reaction, the pH was measured continuously during the reaction time, checking that the initial values were around 11.5. This indicates the alkalinity of the solution, typical of the carbonate zone [23,24]. Throughout the procedure, the solutions were stirred with an electromagnetic magnet at a constant speed of 500 rpm. For the experiments in which temperature and pH value were measured, a thermometer and a pH meter from Trison Instrument (BANDELIN electronic GmbH & Co. KG, Berlin, Germany) were used. The measurements were carried out continuously in order to continually see the changes that occurred. This helped to know when the reaction had finished, at which point the pH value did not change. To perform the experiments well, reproducibility checks were carried out, resulting in an experimental error of ±2% for the regeneration efficiency calculations. The solution was then immediately centrifuged with a centrifuge from J.P. selecta (Barcelona, Spain) at 5000 rpm for 10 min. The obtained supernatant was eliminated and, finally, the solid was taken to the oven, where it was dried for 24 h at 120 °C to obtain a carbonate phase which was characterized by both Raman spectroscopy and Scanning Electron Microscopy (SEM).

As explained before, the parameters studied were molar ratio Ca^2+^/CO_3_^2−^, liquid to solid (L/S) ratio (meaning the mass of water per mass of solid FGD-gypsum) and temperature. Table 2 shows all the experiments carried out in this work, indicating the parameters studied to optimize the results. In order to see how each of these parameters influenced the process, a value was set for each of them and the rest were varied one by one, as performed by previous authors [23,25,26].

Regarding the methodology used for the building material section, carbonated FGD-gypsum and 90 wt% of COMg was prepared in order to analyze the viability of reusing the material as a construction material. The preparation of materials has been completed in accordance with the requirements of the European Standard UNE EN 12859 (CEN, 2012) [27]. Both solid components were introduced into the mixer and stirred for 2 min. The water to solid proportion was 0.5. Once the solids were mixed, the water was poured and the whole mixture was stirred again for 5 min. When a homogeneous and moldable paste was obtained, the mixture was poured into the cylindrical molds (33 mm in diameter and 40 mm in height). Once demolded after casting for 24 h, specimens were cured at 20 °C and 50% humidity for an additional 27 days. A mix with only COMg and the same water/solid ratio was made, in order to compare the effect of the addition of carbonated FGD-gypsum EN 12859 (CEN 2012) [27]. The density of specimens has been determined as an average value among the results obtained from cylinders’ weight and volume measurements. Three samples were evaluated for each composition. The superficial hardness of the samples was determined according to the EN 102031 standard (CEN, 1999) [28] using a Shore C durometer. Five measurements were carried out at different places on each sample. Compressive strength was determined according to the European standard EN 102031 (CEN, 1999) [28]. The Tinius Olsen-TO317EDG test servo-hydraulic machine was used. Three samples were tested.

#### 2.2.2. Physicochemical Characterization

The solid powders obtained had been characterized by different techniques to corroborate the formation of CaCO_3_. The solid CaCO_3_ obtained through filtration was dried at 105 °C for 24 h. The subsequent characterization of the solid was performed using SEM, XRD, and Raman spectroscopy to confirm the formation of CaCO_3_ and to examine its key features. Raman spectroscopy measurements were taken using a Horiba Xplora plus spectrometer ((Horiba Scientific, Kyoto, Japan), equipped with a 532 nm ion laser and a 50x objective. X-ray diffractometer was conducted with a Bruker D8C with a Cu source and θ:θ parallel beam geometry and linear detector. This allows for the study of powders and polycrystalline solids at various temperatures (90–1200 K) and pressures (10–5 to 10 bar) using different inert or reactive atmospheres. To record diffraction patterns, Cu Kα radiation (λ = 0.154 nm) was used. For microstructural characterization, a piece of TENEO equipment (FEI, (Thermo Fisher Scientific, Waltham, MA, USA)) with a field emission scanning electron microscope operated at 5 kV and equipped with an energy-dispersive X-ray spectroscopy system was used. The powders were bathed with a thin layer based on palladium and mounted on pin stubs coated with colloidal graphite-based paint.

#### 2.2.3. Reaction Yield Measurement

**Figure 2:**
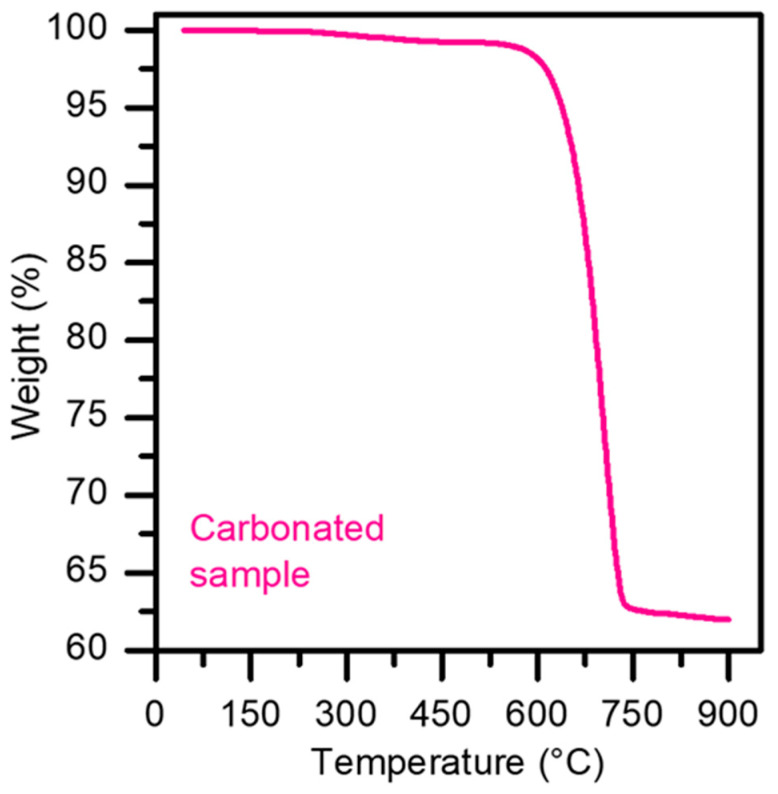
TGA of the carbonated sample.

To measure the reaction yield, thermogravimetric analysis (TGA) was performed, since the loss of mass in the region of 600–800 °C corresponds to CO_2_. For TGA, a high-precision TGA Discovery (TA Instruments) tool measured the mass loss of a material as a function of time and temperature. It features an oven heated by infrared radiation generated by halogen lamps. This technology allows for high heating rates, up to a maximum temperature of 1000 °C in linear mode, with ramp control ranging from 0.1 to 500 °C/min. It includes a mass flow controller module with inputs for up to four different gasses, enabling gas mixing. The operating mode was ramped up from 25 to 950 °C at 20 °C/min in a nitrogen atmosphere. Figure 2 shows an example of the TGA analyses performed, from which the reaction yield was calculated.

## 3. Results and Discussion

This section shows the results of all experiments by varying the molar ratio, the influence of the solution volume, temperature and particle size, as well as the physicochemical characterization of the powders obtained. Finally, the preliminary results of the material used as building material are shown.

### 3.1. Reaction Yield Results

#### 3.1.1. Ca^2+^/CO_3_^2−^ Ratio

**Figure 3:**
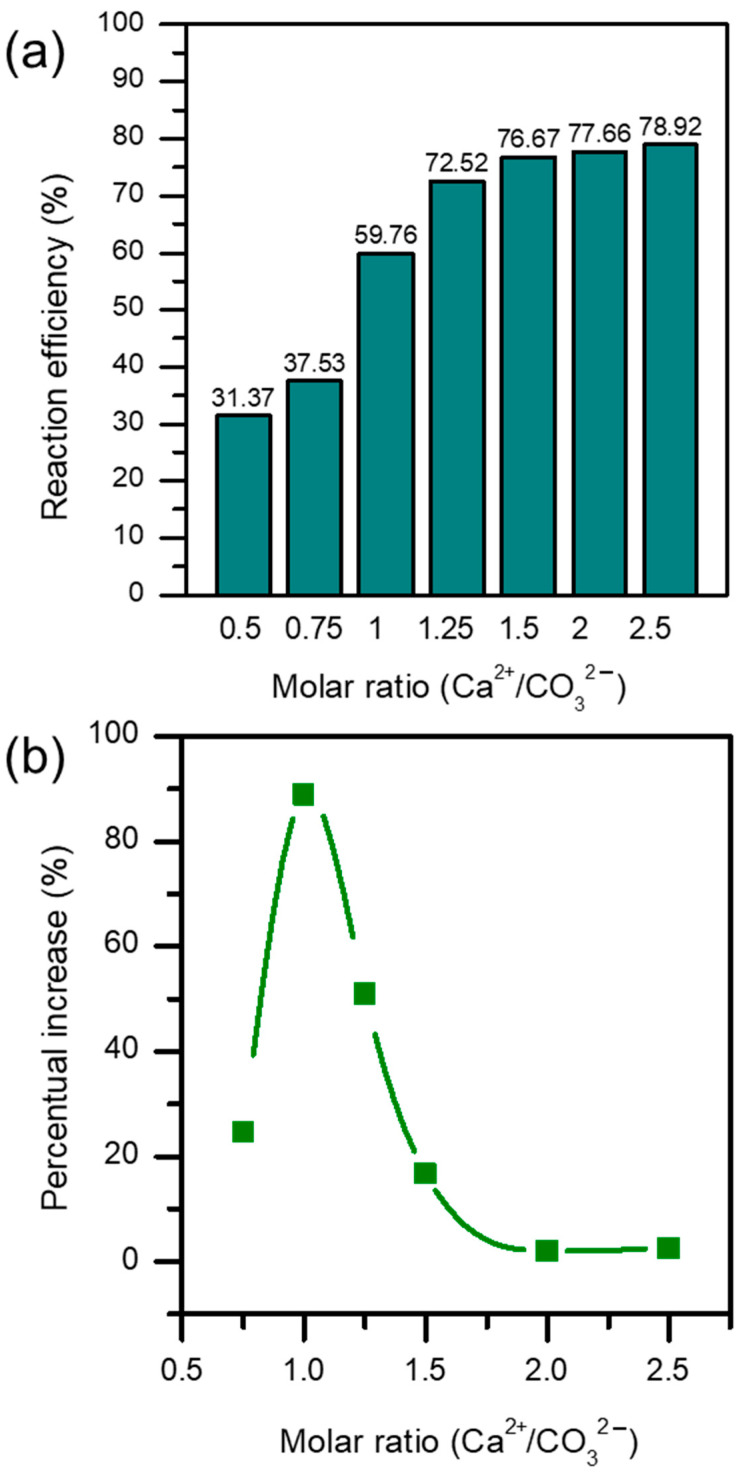
Influence of the molar ratio Ca^2+^/CO_3_^2−^: (**a**) reaction yield; (**b**) percentual increase.

To carry out the optimization of the process, the first parameter to optimize was the molar ratio Ca^2+^/CO_3_^2−^, since it is one of the key factors that influences the performance of the reaction most, as shown previously [16]. Figure 3 reveals the results obtained for the study of this parameter. Figure 3a shows the influence of the obtained reaction yield when the molar ratio was varied. As can be seen, the obtained reaction yield increases considerably as the molar ratio increases, up to the value of 1.25. At this point, the obtained reaction yield is 72.52%. From here, the value increases very slightly as the reaction reaches its maximum yield, probably due to equilibrium-related reasons. For the molar ratio of 2, a reaction yield value of 77.66 is obtained, and for the molar ratio of 2.5 the value is 78.92. In fact, looking at Figure 3b, in which the percentual increase in the reaction yield for each ratio tested is represented, an optimal value point at around a molar ratio of 1 can be seen. However, at a molar ratio of 1, the reaction yield would be 59.76%, meaning that approximately 40% of the calcium would be unreacted. This would lead to very high extra costs in the stages after the precipitation. Hence, a value of 1.25 (72.52% of the reaction yield, which is still a good percentual increase), could be more suitable for the overall economy of the process. In our case, and for the remaining parameters of the study, the value of 1.25 was chosen as the reference, for the reason explained.

#### 3.1.2. L/S Ratio

**Figure 4:**
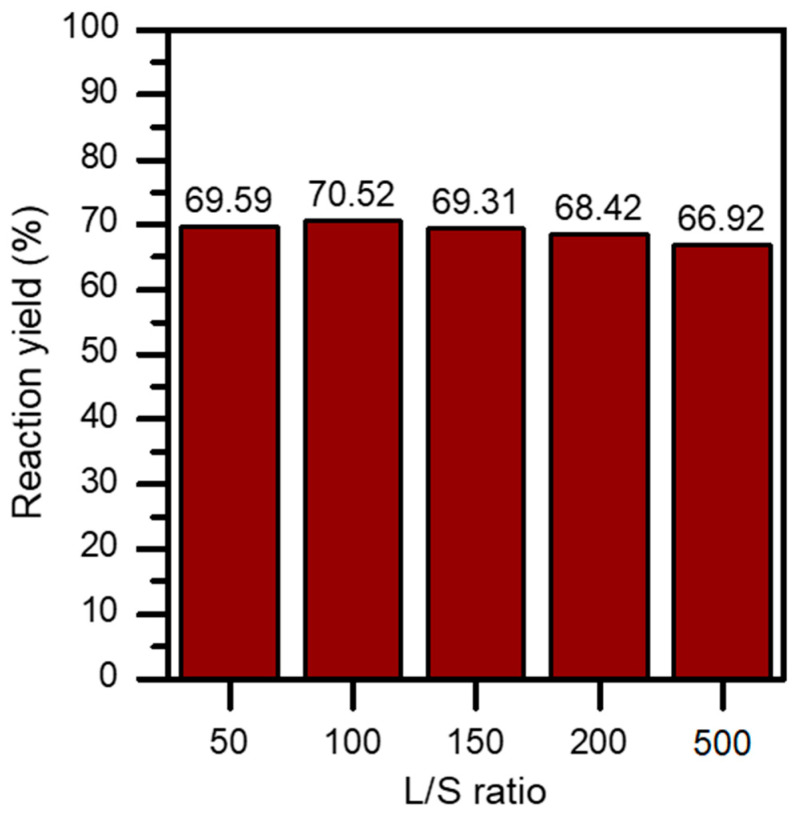
Influence of the L/S ratio on reaction yield.

The L/S ratio was another parameter to be determined in this study, to assess whether this parameter affects the possible interactions that occur during the reaction. In this work, five experiments were carried out, in which the volume of the solution varied between 50 and 500 mL. As can be seen in Figure 4, the effect of the L/S ratio was not decisive for the process studied, since there is no effect on the final result. The results of the tests show that the reaction yield is practically linear. This result is of great interest from an industrial perspective, since the amount of wastewater to be treated after the precipitation process could be drastically reduced. 

#### 3.1.3. Temperature

**Figure 5:**
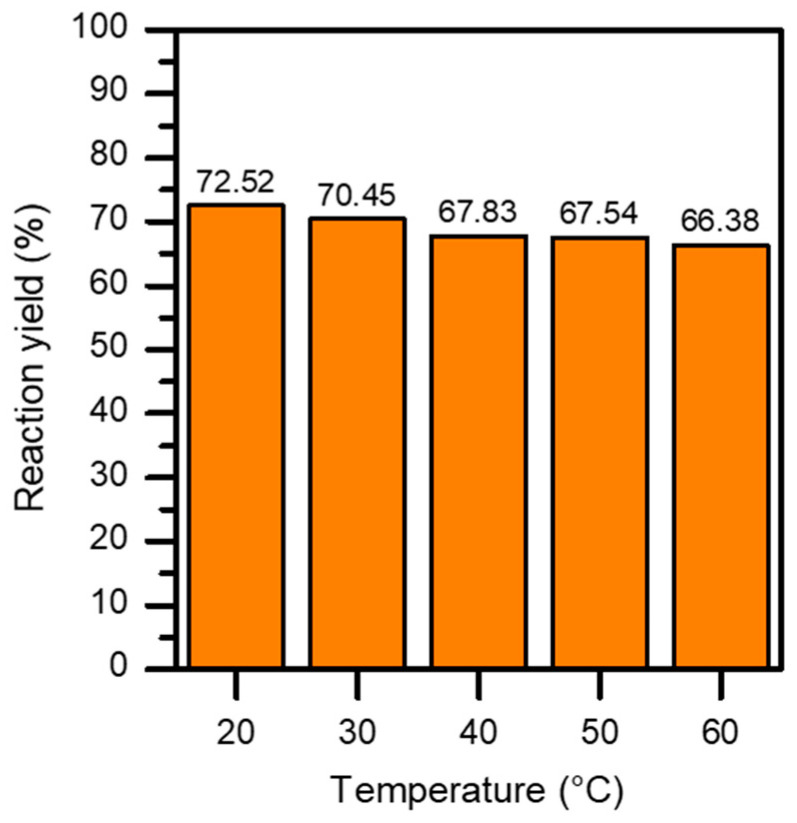
Influence of the temperature on reaction yield.

The influence of temperature was also studied, varying it between 20 °C and 60 °C. Figure 5 shows how temperature influences the reaction yield. As can be seen, the effect of this parameter indicates that there is a slightly linear decreasing trend. This trend could potentially show a direct correlation between the efficiency of the reaction performance and the process temperature. In this case, it can be seen how the temperature negatively influences the performance as it increases, although it is true that the difference is not considerable. In fact, the difference between the most favorable result is less than 7% (72.52% at room temperature versus 66.38% at 60 °C). This results in a lower power consumption during the process compared to other alternatives, such as CO_2_ absorption with monoethanolamine (MEA) [22]. At an industrial level, it is more interesting to work at room temperature than to carry out the process at 60 °C, since it is not necessary to add energy to the process. This is very important in terms of energy efficiency, and is a favorable for the environment. Perhaps this is one of the most interesting results from an energy point of view.

### 3.2. Physicochemical Characterization Results

#### 3.2.1. Raman Measurements

**Figure 6:**
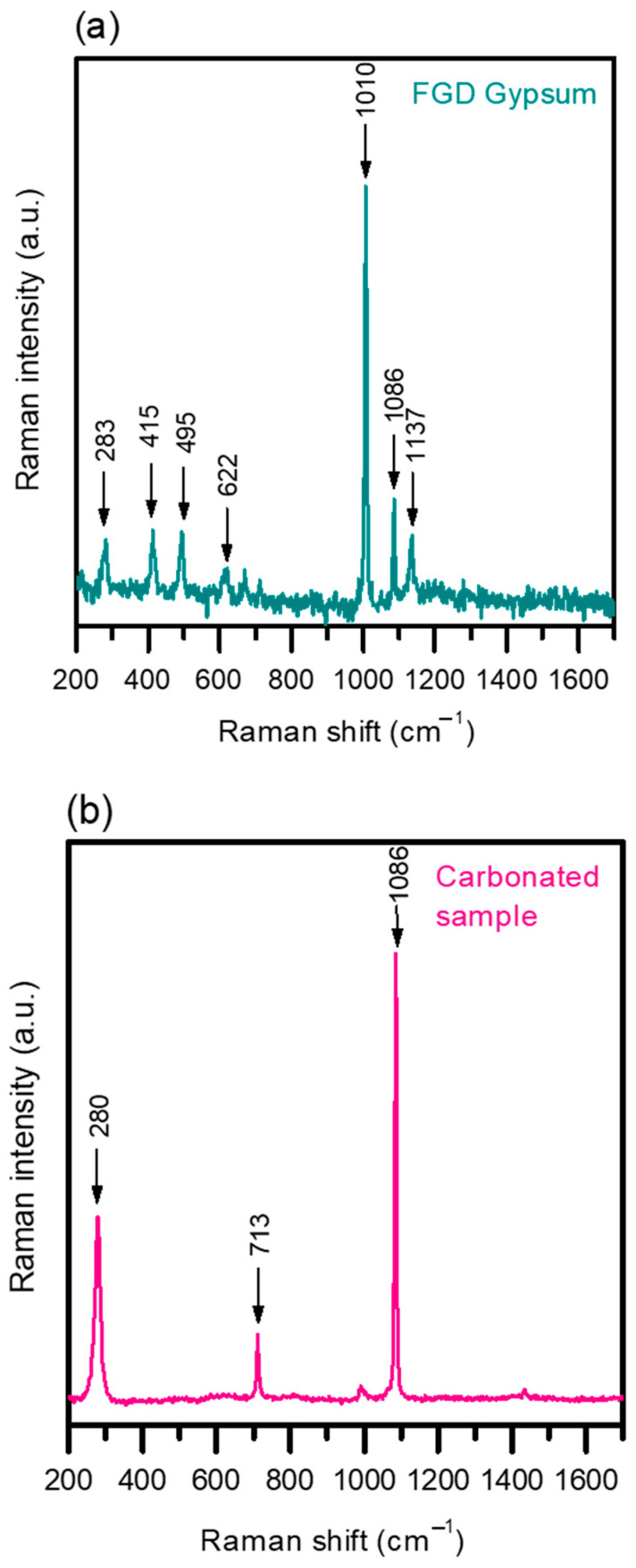
Raman spectra of (**a**) FGD-gypsum and (**b**) carbonated sample.

The physicochemical characterization of the powder samples is essential for assessing the commercial quality of the product and evaluating the process feasibility. Despite all samples yielding identical physicochemical results, this section focuses on the raw gypsum and powders obtained from test 4 for clarity. The initial step involved verifying the presence of carbonates in the samples, for which Raman spectroscopy proved to be a powerful tool in identifying the species within the powder. Figure 6 presents the Raman analysis results for the initial FGD-gypsum and the carbonated sample. In Figure 6a, the waste sample’s typical gypsum composition is evident, with the main band at 1010 cm^−1^ corresponding to the v1 symmetric stretch vibration mode of the SO_4_^2−^ tetrahedra [29], alongside minor bands at 283, 415, 495, 622, 1086 and 1137 cm^−1^, further confirming the gypsum nature of the waste samples [30]. Figure 6b illustrates the Raman spectra of the solid particles from the carbonation experiments, confirming their success. The characteristic CaCO_3_ band at 1086 cm^−1^ is present in the powder from our experiments, matching the monoclinic P21/c group structure [15]. This spectrum is consistent with that of a commercial CaCO_3_, as can be seen in reference [16]. Raman shows three main peaks associated with the carbonate moiety´s vibrations: the v1 symmetric stretch at 1086 cm^−1^, the v4 in-plane bending at 713 cm^−1^, and a lattice mode at 280 cm^−1^, indicative of a calcite structure [31].

#### 3.2.2. XRD Measurements

**Figure 7:**
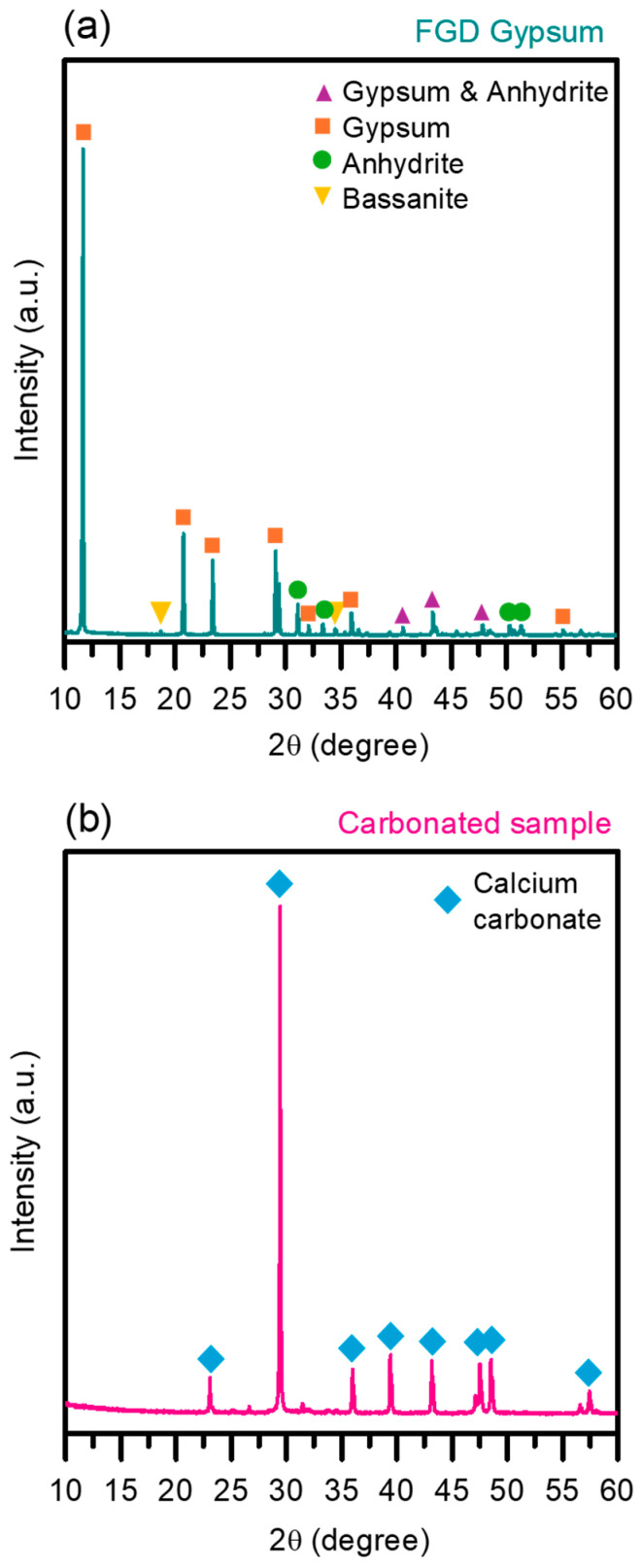
XRD diffractograms of (**a**) FGD-gypsum and (**b**) carbonated sample.

The usability of calcium carbonate is highly dependent on its crystal structure, which can be determined using XRD analysis. Both the FGD-gypsum and the carbonated powders produced in the study were subjected to this technique. Figure 7 displays the results obtained. In particular, Figure 7a shows the XRD patterns of the initial gypsum material, identifying CaSO_4_ in its main morphologies such as anhydrite, bassanite and gypsum, which differ from each other depending on the water content in their structure [32]. Some peaks in anhydrite and gypsum patterns are similar, as noted in Figure 7a, where the waste used as a precipitant exhibits the characteristic features of gypsum as the predominant phase, with some peaks of hydrated polymorphs. For the carbonated sample obtained, Figure 7a presents the XRD pattern. The pattern is identical to a commercial sample [16], confirming the product as calcite, the most stable form of CaCO_3_, characterized by its intense peak at 28° [33]. Calcite has numerous applications, including in the pharmaceutical, cement, paper and polymer industries. Consequently, the carbonate produced in this study shows potential as a valuable industrial product.

#### 3.2.3. SEM Analysis

**Figure 8:**
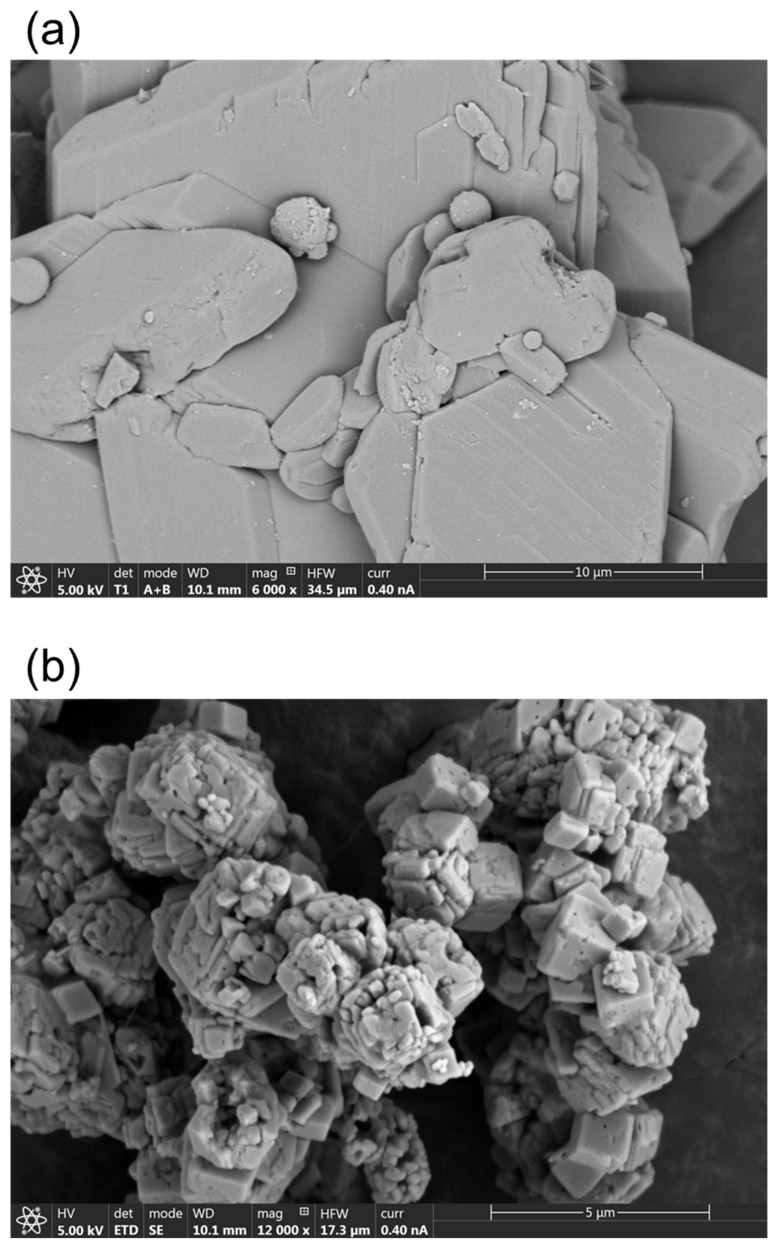
SEM images of (**a**) FGD-gypsum and (**b**) carbonated sample.

SEM is an effective technique for investigating the morphology of solid powders. Figure 8 displays the SEM images of the FGD-gypsum and the carbonated product. As can be seen in Figure 8a, the gypsum obtained has a rhombic structure [12]. In contrast, the final CaCO_3_ product in Figure 8b shows a developed rectangular or rhombohedral morphology characteristic of calcite [16], corroborating the findings from the XRD analysis. These observations confirm the successful transformation and high purity of the final product, ensuring its suitability for various industrial applications. 

### 3.3. Recycling of Wastes in Gypsum Panels

**Figure 9:**
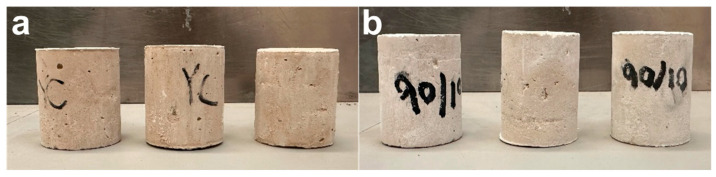
Panels of (**a**) COMg and (**b**) COMg90–C–FGDg10.

Figure 9 shows the commercial gypsum samples and the mixture of commercial gypsum and CO_2_ capture waste. As can be seen, the samples with residues have a greater white color, but in no case is the presence of macropores or efflorescence observed.

Table 3 shows that the variation in the density when the waste is added is negligible, as values exceeding 1100 kg/m^3^ are considered high-density materials in accordance with EN 12859 standard (CEN, 2012) [27]. The specific gravity of commercial gypsum and the waste was calculated using the pycnometer technique. As a result, the waste specific gravity is similar to the commercial gypsum (2.9 and 3.1 g/cm^3^). 

Although the superficial hardness diminishes slightly (5%) when the waste is added, the values for both compositions are all higher than those set by EN (CEN, 2012) [27] for high-density gypsum panels (>80 shore C).

Regarding the compressive strength, the inclusion of the waste reduced its value significantly (20%). Although both compositions exceed the EN 13279 standard’s 2 MPa lower limit (CEN, 2009) [22] for gypsum products, the typical value for commercial gypsum is 8 MPa [34].

## 4. Conclusions and Future Remarks

In this work, flue gas desulfurization gypsum has been used as a material to capture CO_2_ and, in addition, to use the CaCO_3_ produced by the process as a construction material. Thus, a waste product is revalued to improve the environmental footprint. The results obtained show, on a laboratory scale, the good performance of the reaction. The Ca/CO_3_ ratio of 1.25 (72.52% reaction yield) proved to be more suitable for the overall economy of the process. As for the L/S ratio, 100 mg/L was the optimum. In this case, the reaction volume did not vary much in the results, which is of great interest on an industrial scale, since the amount of wastewater to be treated after the precipitation process can be reduced. Finally, the temperature parameter showed that the process gives good results at room temperature. These data are of vital importance, because the process can be carried out without adding energy, helping the environment in terms of energy efficiency. Furthermore, the results were characterized by Raman, XRD and SEM, confirming the presence of CaCO_3_ and its main phase, as well as the morphology of the product obtained.

Furthermore, the waste after the CO_2_ capture was used as a gypsum-based construction material, measuring the density, surface hardness and compressive strength of a material composed of a 10% weight of CaCO_3_ and a 90% weight of commercial plaster, with a water/solid ratio of 0.5. As a result, both the density and the mechanical properties were reduced when the waste was added, but the values are higher than the mechanical requirements for this product. 

The most important added value of this research is considered to be the answer to the following questions: (1) What is the optimal Ca^2+^/CO_3_^2−^ ratio for the reaction? (2) How does the liquid to solid ratio influence the carbonation? (3) What role does the temperature play here? These questions are key for the industrial development of waste to added-value processes. Furthermore, another added value of our work can be found in the utilization of the produced CaCO_3_ as a building material, closing the circle from a circular economy perspective. The authors would like to highlight some limitations of this study, including the following: (1) the CO_2_ capture stage is not studied, since it has been previously studied by several researchers; (2) the effect of some parameters on the reaction yield, such as, for example, the stirring rate or reactor shape type, have not been included; (3) the building properties of the material produced are examined only in a preliminary manner, and further experiments should be carried out to corroborate some of the important properties. All of these points will be further studied in future works.

## Figures and Tables

**Table 1:** Chemical composition of FGD-gypsum and COMg.

	FGD-Gypsum	COMg
Humidity (Loss at 105 °C)	26.5	6.1
Loss at 750 °C	7.7	9.1
Fe_2_O_3_ (%)	0.1	0.3
CaO (%)	44.5	43.7
MgO (%)	0.4	2.0
SiO_2_ (%)	1.1	3.2
Al_2_O_3_ (%)	0.3	0.7
Na_2_O (%)	0.02	0.07
K_2_O (%)	0.1	0.2
SO_3_ (%)	53.5	49.9

**Table 2:** Parameters studied in carrying out the experiments.

Test	Molar Ratio Ca^2+^/CO_3_^2−^	L/S Ratio	Temperature (°C)
1	0.5	100	25
2	0.75	100	25
3	1	100	25
4	1.25	100	25
5	1.5	100	25
6	2	100	25
7	2.5	100	25
8	1.25	50	25
9	1.25	150	25
10	1.25	200	25
11	1.25	500	25
12	1.25	100	20
13	1.25	100	30
14	1.25	100	40
15	1.25	100	50
16	1.25	100	60

**Table 3:** Physical and mechanical properties of COMg and COMg90-C-FGDg10.

Composition	Density (kg/m^3^)	Superficial Hardness (Shore C)	Compressive Strength (MPa)
COMg	1338 ± 19	90.6 ± 3	7.8 ± 0.9
COMg90–C–FGDg10	1326 ± 21	86.1 ± 2	6.1 ± 0.6

## Data Availability

All data used to support the findings of this study are available from the corresponding author upon request.
